# Amygdala opioid receptors mediate the electroacupuncture-induced deterioration of sleep disruptions in epilepsy rats

**DOI:** 10.1186/1423-0127-20-85

**Published:** 2013-11-12

**Authors:** Pei-Lu Yi, Chin-Yu Lu, Chiung-Hsiang Cheng, Yi-Fong Tsai, Chung-Tien Lin, Fang-Chia Chang

**Affiliations:** 1 Department of Veterinary Medicine, School of Veterinary Medicine, National Taiwan University, Taipei, Taiwan; 2 Department of Sports, Health & Leisure, College of Sports Knowledge, Aletheia University, Tainan Campus, Taiwan; 3 Graduate Institute of Brain & Mind Sciences, College of Medicine, National Taiwan University, Taipei, Taiwan; 4 Graduate Institute of Acupuncture Science, College of Chinese Medicine, China Medical University, Taichung, Taiwan

**Keywords:** Electroacupuncture, Feng-Chi (GB20), Epilepsy, Amygdala, Sleep, Opioid receptors

## Abstract

**Background:**

Clinical and experimental evidence demonstrates that sleep and epilepsy reciprocally affect each other. Previous studies indicated that epilepsy alters sleep homeostasis; in contrast, sleep disturbance deteriorates epilepsy. If a therapy possesses both epilepsy suppression and sleep improvement, it would be the priority choice for seizure control. Effects of acupuncture of Feng-Chi (GB20) acupoints on epilepsy suppression and insomnia treatment have been documented in the ancient Chinese literature, Lingshu Jing (Classic of the Miraculous Pivot). Therefore, this study was designed to investigate the effect of electroacupuncture (EA) stimulation of bilateral Feng-Chi acupoints on sleep disruptions in rats with focal epilepsy.

**Results:**

Our result indicates that administration of pilocarpine into the left central nucleus of amygdala (CeA) induced focal epilepsy and decreased both rapid eye movement (REM) sleep and non-REM (NREM) sleep. High-frequency (100 Hz) EA stimulation of bilateral Feng-Chi acupoints, in which a 30-min EA stimulation was performed before the dark period of the light:dark cycle in three consecutive days, further deteriorated pilocarpine-induced sleep disruptions. The EA-induced exacerbation of sleep disruption was blocked by microinjection of naloxone, μ- (naloxonazine), κ- (*nor*-binaltorphimine) or δ-receptor antagonists (natrindole) into the CeA, suggesting the involvement of amygdaloid opioid receptors.

**Conclusion:**

The present study suggests that high-frequency (100 Hz) EA stimulation of bilateral Feng-Chi acupoints exhibits no benefit in improving pilocarpine-induced sleep disruptions; in contrast, EA further deteriorated sleep disturbances. Opioid receptors in the CeA mediated EA-induced exacerbation of sleep disruptions in epileptic rats.

## Background

Clinical and experimental observations have demonstrated that sleep and epilepsy reciprocally affect each other. While rapid eye movement (REM) sleep suppresses seizure activity, non-REM (NREM) sleep facilitates it [1,2]. On the other hand, patients with epilepsy experience more daytime sleepiness compared with control patients [3], and children with epilepsy experience poor quality of sleep, anxiety about sleep, and sleep-disordered breathing [4]. Our previous studies have demonstrated that occurrence of epilepsy at different zeitgeber time points results in different sleep disruptions by altering either the homeostatic factors or circadian rhythm of the sleep-wake regulation in rats [5,6]. Notably, epilepsy-induced sleep disruptions further deteriorate and worsen seizure control [7]. Therefore, if a therapy possesses both epilepsy suppression and improvement of sleep disturbance, it becomes the most appropriate therapy for seizure control.

Acupuncture has been used as an alternative medicine to treat patients with chronic pain [8] (e.g., migraines, tension-type headache and peripheral joint osteoarthritis [9]) by manipulating thin needles and inserting into acupoints. Electroacupuncture (EA) is a modified form of acupuncture, which is performed by passing an electrical current through needles into the acupoints and controls the acupuncture dose by modifying the frequency and intensity of stimulating currents. Acupuncture had been documented in some Chinese literatures indicating the therapeutic effect in epilepsy, insomnia, and other neurological diseases, in addition to pain relief. For example, the acupoints of Feng-Chi (GB20) has indications of epileptic suppression and insomnia, documented in the Lingshu Jing (Classic of the Miraculous Pivot). However, there is a lack of scientific evidence to elucidate its underlying mechanism and to prove the clinical efficacy. Our observation demonstrated that 100 Hz EA stimulation of bilateral Feng-Chi acupoints exacerbates focal epilepsy induced by administering pilocarpine into the left central nucleus of amygdala (CeA). However, the effect of 100 Hz EA stimulation of bilateral Feng-Chi acupoints on sleep disturbances in rats with focal epilepsy has not been investigated. Previous studies have demonstrated that EA stimulation of bilateral Anmian (EX17) acupoints enhances sleep through the activation of vagus nerve, which subsequently activates opioid receptors in the nucleus of tractus solitarius (NTS) [10,11]. Feng-Chi acupoint is anatomically close to the Anmian acupoint. It has been indicated that amygdala receives the afferent projection from the NTS [12,13]. Altering NTS activity changes dynorphin gene expression in the amygdala [14]. Furthermore, amygdala plays an important role in the sleep regulation [15]–[18], especially in REM sleep [16]–[18]. Therefore, stimulation of Feng-Chi acupoints may activate vagus nerve and subsequently modify the opioid receptors in the amygdala to achieve its effect in the sleep-wake regulation. This current study was designed to elucidate the effect of high-frequency (100 Hz) EA stimulation of Feng-Chi acupoints in the sleep alterations induced by the amygdaloid focal epilepsy.

Discovery of endogenous opioid peptides, including β-endorphin, dynorphin, enkephalin and endomorphin, in the central nervous system (CNS) reveals the mysterious actions of acupuncture, especially in its analgesic effect [19]–[21]. Han and his colleagues have shown that endogenous opioids and distinct opioid receptors mediate the analgesic effect induced by both low-frequency and high-frequency EA stimuli [22,23]. While μ- and δ-opioid receptors in the spinal cord are dominant in the low-frequency EA-induced analgesia, κ-opioid receptors contribute to the high-frequency EA effect [22,23]. Our previous results also delineated that 10 Hz EA stimulation of Anmian acupoints enhances NREM sleep through activation of μ-opioid receptors in the caudal NTS [10], whereas κ-receptors contribute to the 100 Hz EA-induced sleep enhancement [11]. This study further elucidated the involvement of CeA opioid receptors in sleep alterations induced by high-frequency (100 Hz) EA stimulation of bilateral Feng-Chi acupoints in rats with amygdaloid focal epilepsy.

## Methods

### Pharmacological agents

Stock solutions of a broad-spectrum opioid antagonist (naloxone hydrochloride (Tocris, Bristol, UK)), a μ-receptor antagonist (naloxonazine dihydrochloride (Tocris)), a δ-receptor antagonist (naltrindole hydrochloride (Tocris)) and a κ-receptor antagonist (*nor*-binaltorphimine dihydrochloride (Tocris)) were dissolved in pyrogen-free saline (PFS). Pilocarpine (1 mg/1 μl, Sigma-Aldrich, St. Louis, MO, USA) was also dissolved in PFS. The stock solutions were stored at 4°C until use. Our previous results and others indicated that the appropriate microinjection dosage for naloxonazine, naltrindole and *nor*-binaltorphimine to selectively block μ-, δ- and κ-opioid receptors, without interaction with other opioid receptor subtypes, is within 20 μg [10,11,24,25]. In current study, naloxone, naloxonazine, naltrindole and *nor*-binaltorphimine were microinjected at the dose of 10 μg/μl, which efficiently exhibits their specific pharmacological blockade according to our previous studies [10,11]. The total volume for each injection was 1 μl and the duration of injection was 3 to 5 minutes. Our previous study has demonstrated that microinjection of 1 μl solution into the CeA did not cause CeA lesion [26].

### Animals

Male Sprague-Dawley rats (250 - 300 g, 8-9 week old; National Laboratory Animal Breeding and Research Center, Taiwan) were used in this study. Rats were anesthetized by intraperitoneal injection with 50 mg/kg Zoletil® (Virbac, Carros, France). Rats were surgically implanted with three electroencephalogram (EEG) screw electrodes as previously described [27] and a microinjection guide cannulae directed into the left CeA (AP, 2.8 mm from bregma; ML, 4.2 mm; DV, 7.8 mm relative to bregma). The coordinates were adopted from the Paxinos and Watson rat atlas [28]. Two screw EEG electrodes were placed over the left frontal and parietal lobes of cortices, and a third EEG electrode was placed over the right cerebellum and served to ground the animal to reduce signal artifacts. Insulated leads from EEG electrodes were routed to a Teflon pedestal (Plastics One, Roanoke, VA, USA). The Teflon pedestal was then cemented to the skull with dental acrylic (Tempron, GC Co., Tokyo, Japan). The incision was treated topically with polysporin (polymixin B sulfate – bacitracin zinc) and the animals were allowed to recover for seven days prior to the initiation of experiments. The rats were housed separately in individual recording cages in the isolated room, in which the temperature was maintained at 23 ± 1°C and the light:dark (L:D) rhythm was controlled in a 12:12 h cycle (40 Watt x 4 tubes illumination). Food (5001 rodent diet, LabDiet) and water were available *ad libitum*. All procedures performed in this study were approved by the National Taiwan University Animal Care and Use Committee. (IACUC Approval number: 98 Experiment Approval No. 95).

### Experimental protocol

On the 2^nd^ postsurgical day, the rats were connected to the recording apparatus (see below) via a flexible tether. As such, rats were allowed relatively unrestricted movement within their own cages. One week after rats had adapted to the 12:12-hour L:D cycle after surgery, a 24-hour undisturbed baseline of the sleep-wake activity, determined by the EEG and gross body movement (see later), were obtained beginning at dark onset on the 1^st^ recording day in rats from all groups. Nine groups of rats were used in the study as follows. Rats in group 1 (n = 6) received a 30-min 100 Hz EA stimulation of bilateral Feng-Chi acupoints per day, beginning at 30 minutes before the dark period and performing in three consecutive days (the EA group). Sleep-wake activity was recorded right after the end of the last period of EA stimuli and lasted for 24 hours. Rats in group 2 (n = 6) were administered with pilocarpine into the left CeA and the sleep-wake activity was recorded beginning from the dark onset of the L:D cycle (the pilocarpine group). In group 3 (n = 6), rats received the same EA stimulation protocol as those rats in group 1 and were respectively administered with PFS and pilocarpine into the CeA before and after the last period of EA stimulation (the PFS + EA + pilocarpine group). Rats in group 4 (n = 6) were used to determine the effects of opioid receptor antagonist, naloxone, on the 100 Hz EA-induced sleep alterations in rats with focal epilepsy (the naloxone + EA + pilocarpine group). Rats in groups 5 (n = 6), 6 (n = 6), and 7 (n = 6) were respectively used to depict the effects of μ-receptor antagonist (naloxonazine, the naloxonazine + EA + pilocarpine group), δ-receptor antagonist (naltrindole, the natrindole + EA + pilocarpine group) and κ-receptor antagonist (*nor*-binaltorphimine, the *nor*-binaltorphimine + EA + pilocarpine group) on the 100 Hz EA-induced sleep alterations in rats with focal epilepsy. Rats in groups 4-7 received the similar protocol as those in group 3, except that naloxone (10 μg/μl), naloxonazine (10 μg/μl), naltrindole (10 μg/μl) and *nor*-binaltorphimine (10 μg/μl) were administered into the CeA before the last period of EA stimulation in groups 4, 5, 6 and 7, respectively. Rats in group 8 (n = 6) had the similar protocol as those in group 3, except that rats received the sham EA stimulation (the sham EA group, the sham EA stimulation described later). Rats in group 9 (n = 9), used as the control group, received PFS, naloxone, naloxone + EA and naloxone + pilocarpine at the dark onset in three consecutive days. When 100 Hz EA was given (see later), all rats were lightly anesthetized with 29/4 mg/kg of ketamine/xylazine (one third of the dose which we used for surgery in our previous studies [10,11,27]), after which rat woke up in 30 to 35 minutes. A 30-min period of EA stimulation was administered before the onset of the dark period per day and was applied in three consecutive days. The anesthetization was given 30 minutes prior to the dark period and lasted for 30 minutes. The 100 Hz EA stimulus was delivered via the bilateral insertion of stainless needles (32 gauge x 1”, Shanghai Yanglong Medical Articles Co.) on Feng-Chi (GB20) acupoints in the depth of 2 mm. The stimulus consisted of a train of biphasic pulses (150 μs duration each) of 100 Hz with intensity of 1 mA, and was delivered by Functions Electrical Stimulator (Trio 300, I.T.O., Japan). The EA stimulation parameters are the same as we used in the previous study [11]. The location of Feng-Chi acupoints in the rat was determined by the same anatomical location described in human. The acupoint of Feng-Chi (GB 20) locates in the depression between the upper portion of m. sternocleidomastoideus and m. trapezius in human. In rats, Feng-Chi acupoint locates 3 mm away from the center of a line between two ears [29]. Sham EA was performed by stimulation of a non-acupoint located at the ventral conjunction between the forelimb and the trunk as previously described [30]. Rats, anesthetized by ketamine/xylazine, received same electrical stimuli, including same intensity and frequency, but the stimulation site did not locate at any acupoint.

### Apparatus and recording

Signals from the EEG electrodes were fed into an amplifier (Colbourn Instruments, Lehigh Valley, PA; model V75-01). The EEG was amplified (factor of 5,000) and analog bandpass filtered between 0.1 and 40 Hz (frequency response: ±3 dB; filter frequency roll off: 12 dB / octave). Gross body movements were detected by custom-made infrared-based motion detectors (Biobserve GmbH, Germany), and the movement activity was converted to a voltage output, which was digitized and integrated into 1-s bins. These conditioned signals (EEGs and gross body movements) were subjected to analog-to-digital conversion with 16-bit precision at a sampling rate of 128 Hz (NI PCI-6033E; National Instruments, Austin, TX). The digitized EEG waveform and integrated values for body movement were stored as binary computer files pending subsequent analyses.

Postacquisition determination of the vigilance state was done by visual scoring of 12-s epochs using custom software (ICELUS, Mark R. Opp) written in LabView for Windows (National Instruments). The animal’s behavior was classified as either NREM sleep, REM sleep or waking based on previously defined criteria [27]. Briefly, NREM sleep is characterized by large-amplitude EEG slow waves, high power density values in the delta frequency band (0.5 – 4.0 Hz), and lack of gross body movements. During REM sleep, the amplitude of the EEG is reduced, the predominant EEG power density occurs within the theta frequency (6.0 – 9.0 Hz) and there are phasic body twitches. During waking, the rats are generally active. There are protracted body movements. The amplitude of the EEG is similar to that observed during REM sleep, but power density values in the delta frequency band are generally greater than those in theta frequency band.

### Statistical analyses for experiment protocol

All values acquired from sleep-wake recording were presented as the mean ± SEM for the indicated sample sizes. One-way ANOVA for the duration of each vigilance state (NREM sleep, REM sleep, WAKE) was performed, comparing the effects of different manipulations between groups, across the certain of time block. If statistically significant differences were detected, a Fisher’s post-hoc comparison was made to determine which values during experimental conditions deviated from those obtained from the control condition. An α level of p < 0.05 was considered as indicating a statistically significant difference.

## Results

### The effect of 100 Hz EA stimulation of bilateral Feng-Chi acupoints on sleep in normal rats

**Figure 1:**
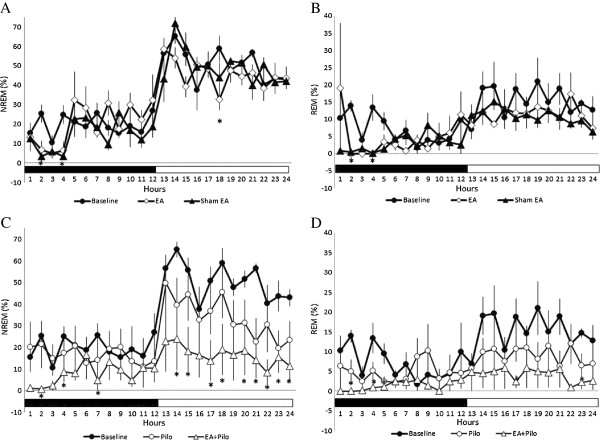
**The effects of 100 Hz EA stimulation and pilocarpine in sleep.** Panels **A** &**B**: NREM sleep and REM sleep obtained from the undisturbed baseline, shame EA group and EA group. Panel **C** &**D**: NREM sleep and REM sleep acquired from the baseline, the pilocarpine group and the PFS + EA + pilocarpine group. Black circles: the values obtained from undisturbed rats (baseline); black triangles: the values obtained from the sham EA group; open diamonds: the values acquired from the EA group; open circles: the values obtained from the pilocarpine group; open triangles: the data acquired from the PFS + EA + pilocarpine group. *: p < 0.05 vs. baseline. Black bar: the dark period; white bar: the light period.

**Figure 2:**
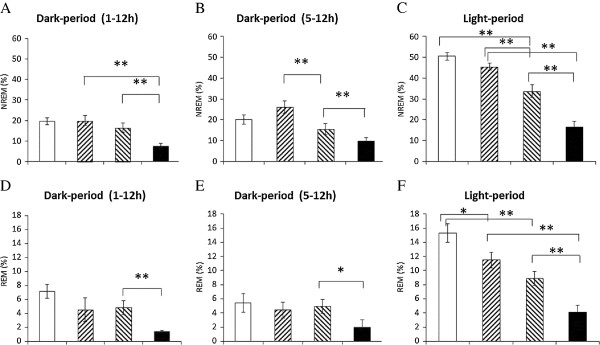
**The summary of the effects of 100 Hz EA stimulation and pilocarpine in sleep.** The bars from the left to the right in each panel represent the data acquired from the baseline, the EA group, the pilocarpine group and the PFS + EA + pilocarpine group. Panels **A**, **B** and **C** represent the percentage of time spent in NREM sleep during the 12-h dark period, the time block between 5-12 h of the dark period, and the 12-h light period, respectively. Panels **D**, **E** and **F** indicate the percentage of time spent in REM sleep during the 12-h dark period, the time block between 5-12 h of the dark period, and the 12-h light period, respectively. **: p < 0.01.

High-frequency (100 Hz) EA stimulation of bilateral Feng-Chi acupoints did not significantly alter both NREM sleep and REM sleep during the dark period (Figure 1A, 1B & 2A), except that there were decreases of NREM sleep and REM sleep during the first 4 hours of the dark period after EA stimuli. Our previous results have demonstrated that anesthetization of rats for 30 minutes with ketamine, an NMDA receptor antagonist, prior to the dark period suppressed both NREM sleep and REM sleep during the first few hours of the dark period [10,30]. Therefore, the decreases of NREM sleep and REM sleep during the first 4 hours of the dark period, when rats received the 100 Hz EA stimulation of bilateral Feng-Chi acupoints and were under anesthetization (the EA group), were primarily due to the effect of ketamine (Figure 1A & 1B). Our previous results have also demonstrated that EA stimulation of bilateral Anmian acupoints under the same method as this current experimental design enhances sleep during the hours 5-12 of the dark period [10,11,30]. We further compared the values obtained from rats received 100 Hz EA of Feng-Chi acupoints with that acquired from undisturbed baseline during the hours of 5-12 of the dark period, and found there was no statistically significant changes in both NREM sleep and REM sleep (Figure 2B & 2E). The percentage of time spent in NREM sleep during hours 5-12 obtained from the undisturbed baseline and from the rats with EA stimuli was 20.0 ± 2.2% and 26.1 ± 3.0% (p > 0.05; Figure 2B), respectively. Nevertheless, REM sleep during the following light period was decreased from 15.3 ± 1.3% obtained from the undisturbed baseline to 11.5 ± 1.1% acquired from rats treated with 100 Hz EA stimuli (p < 0.05, Figure 2F). There was no change, except that the sleep suppression caused by ketamine during the first 4-h post-administration, in the sleep-wake activity when rats received the sham EA stimulation (the sham group, Figure 1A & 1B), which is consistent with our previous results [11,30]. This observation indicated that high-frequency EA stimulation of bilateral Feng-Chi acupoints did not alter NREM sleep, but decreased REM sleep during the light period in normal rats.

### The effect of administration of pilocarpine into the left CeA on sleep

Administration of pilocarpine into the left CeA induced focal epilepsy, which has been validated by analyzing epileptiform EEGs recorded from the frontal, parietal, occipital cortices of the left and right hemispheres in our preliminary study. The occurrences of epileptiform EEGs were observed during the dark period and during the following light period. Administration of pilocarpine into the CeA at the beginning of the dark period did not significantly change the amounts of NREM sleep and REM sleep during the dark period; however, both NREM sleep and REM sleep were significantly decreased during the following 12-h light period (Figure 1C & 1D). The percentage of time spent in NREM sleep during the light period was decreased from 50.5 ± 1.9% obtained from undisturbed rats to 33.8 ± 3.2% acquired after administration of pilocarpine (the pilocarpine group) at the dark onset (p < 0.05, Figure 2C), and the amount of REM sleep during the light period was also reduced from 15.3 ± 1.3% to 8.9 ± 1.0% (p < 0.05, Figure 2F).

### The effect of 100 Hz EA stimulation of bilateral Feng-Chi acupoints on pilocarpine-induced sleep alterations

Rats received a 30-min 100 Hz EA stimulation of bilateral Feng-Chi acupoints in three consecutive days did not prevent pilocarpine-induced sleep reduction; in contrast, it worsened the sleep disruptions (Figure 1C & 1D). The percentage of time spent in NREM sleep during the dark period was significantly decreased from 16.5 ± 2.2% obtained from the pilocarpine group to 7.4 ± 1.4% acquired from the PFS + EA + pilocarpine group (p < 0.05, Figure 2A), and the amount of REM sleep was also significantly reduced from 4.8 ± 1.0% to 1.4 ± 0.1% (p < 0.05, Figure 2D). The percentage of time spent in NREM sleep during the light period was significantly decreased from 33.8 ± 3.2% obtained from the pilocarpine group to 16.4 ± 2.8% acquired from the PFS + EA + pilocarpine group (p < 0.05, Figure 2C), and the amount of REM sleep was also significantly reduced from 8.9 ± 1.0% to 4.1 ± 1.0% (p < 0.05, Figure 2F).

### The effect of naloxone on the 100 Hz EA-induced sleep alterations

**Figure 3:**
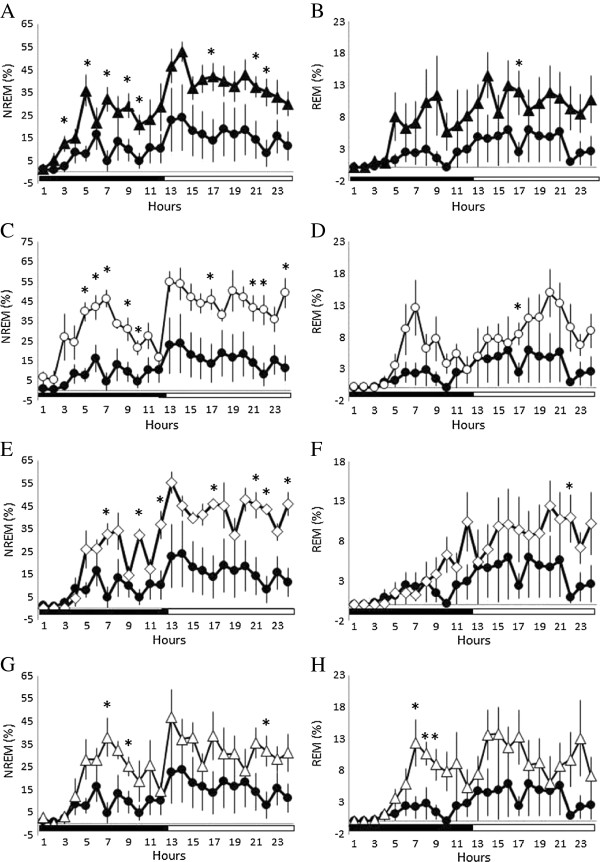
**The effects of naloxone, naloxonazine, naltrindole and *nor*-binaltorphimine on the EA-induced exacerbation of sleep disruptions in rats with focal epilepsy.** Panels **A** &**B**: the effects of naloxone on EA-induced disruptions of NREM sleep and REM sleep. Panels **C** &**D**: the effects of naloxonazine on EA-induced disturbances of NREM sleep and REM sleep. Panels **E** &**F**: the effects of naltrindole on EA-induced disruptions of NREM sleep and REM sleep. Panels **G** &**H**: the effects of *nor*-binaltorphimine on EA-induced disturbances of NREM sleep and REM sleep. Black circles: the data obtained from the PFS + EA + pilocarpine group; black triangles: the values acquired from the naloxone + EA + pilocarpine group; open circles: the results obtained from the naloxonazine + EA + pilocarpine group; open diamonds: the data acquired from the naltrindole + EA + pilocarpine group; open triangles: the values obtained from the *nor*-binaltorphimine + EA + pilocarpine group. *: p < 0.05 vs. the values obtained from the PFS + EA + pilocarpine group. Black bar: the dark period; white bar: the light period.

**Figure 4:**
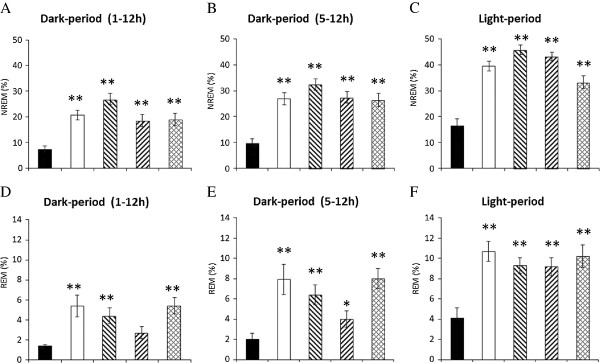
**The summary of the effects of naloxone**, **naloxonazine**, **naltrindole and *nor***-**binaltorphimine on the EA**-**induced exacerbation of sleep disruptions**. Panels **A**, **B** and **C** demonstrate the percentage of NREM sleep acquired from the indicated time blocks. Panels **D**. **E** and **F** depict the percentage of REM sleep obtained from the indicated time blocks. The bars from the left to the right in each panel represent the data acquired from the PFS + EA + pilocarpine group, the naloxone + EA + pilocarpine group, the naloxonazine + EA + pilocarpine group, the naltrindole + EA + pilocarpine and the *nor*-binaltorphimine + EA + pilocarpine group. Panels **A**, **B** and **C** represent the percentage of time spent in NREM sleep during the 12-h dark period, the time block between 5-12 h of the dark period, and the 12-h light period, respectively. Panels **D**, **E** and **F** indicate the percentage of time spent in REM sleep during the 12-h dark period, the time block between 5-12 h of the dark period, and the 12-h light period, respectively. **: p < 0.01 vs. the values acquired from the PFS + EA + pilocarpine group.

Administration of naloxone significantly blocked the 100 Hz EA-induced reductions of NREM sleep and REM sleep during both the dark period and light period (Figure 3A & 3B). The percentage of time spent in NREM sleep and REM sleep during the dark period obtained from the naloxone + EA + pilocarpine group significantly increased to 20.7 ± 2.0% (p < 0.05, when compared with the values obtained from the PFS + EA + pilocarpine group) and 5.4 ± 1.1% (p < 0.05, when compared with the values obtained from the PFS + EA + pilocarpine group), respectively (Figure 4A & 4D). The percentage of time spent in NREM sleep and REM sleep during the light period obtained from the naloxone + EA + pilocarpine group significantly increased to 39.4 ± 1.8% (p < 0.05, when compared with the values obtained from the PFS + EA + pilocarpine group) and 10.7 ± 1.0% (p < 0.05, when compared with the values obtained from the PFS + EA + pilocarpine group), respectively (Figure 4C & 4F). Our results also demonstrated that administration of naloxone into the CeA did not change sleep activities in naïve rats. The percentage of time spent in NREM sleep during the dark period obtained from rats received PFS and naloxone was 18.6 ± 1.6% and 21.6 ± 1.5% (p = 0.160), respectively. The percentage of time spent in REM sleep during the dark period obtained from rats received PFS and naloxone was 6.0 ± 0.8% and 5.2 ± 0.7% (p = 0.474), respectively. The percentage of time spent in NREM sleep during the light period obtained from rats received PFS and naloxone was 45.9 ± 1.7% and 46.1 ± 1.6% (p = 0.921), respectively. The percentage of time spent in REM sleep during the light period obtained from rats received PFS and naloxone was 7.4 ± 0.7% and 8.1 ± 0.8% (p = 0.531), respectively. Naloxone also exhibited no significant effect on sleep-wake parameters obtained either after EA stimuli or pilocarpine administration (data not shown). These observations suggest that the EA-induced exacerbation of sleep disruptions was specifically mediated by opioid receptors.

### The effect of naloxonazine on the 100 Hz EA-induced sleep alterations

Administration of naloxonazine exhibited a similar effect to that of naloxone in blocking the 100 Hz EA-induced reductions of NREM sleep and REM sleep during both the dark period and light period (Figure 3C & 3D). The percentage of time spent in NREM sleep and REM sleep during the dark period obtained from the naloxonazine + EA + pilocarpine group significantly increased to 26.9 ± 2.2% (p < 0.05, when compared with the values obtained from the PFS + EA + pilocarpine group) and 4.4 ± 0.8% (p < 0.05, when compared with the values obtained from the PFS + EA + pilocarpine group), respectively (Figure 4A & 4D). The percentage of time spent in NREM sleep and REM sleep during the light period obtained from the naloxonazine + EA + pilocarpine group significantly increased to 45.7 ± 1.9% (p < 0.05, when compared with the values obtained from the PFS + EA + pilocarpine group) and 9.3 ± 0.8% (p < 0.05, when compared with the values obtained from the PFS + EA + pilocarpine group), respectively (Figure 4C & 4F).

### The effect of naltrindole on the 100 Hz EA-induced sleep alterations

Administration of naltrindole also exhibited a similar effect to those of naloxone and naloxonazine in blocking the 100 Hz EA-induced reductions of NREM sleep and REM sleep during both the dark period and light period (Figure 3E & 3F). The percentage of time spent in NREM sleep during the dark period obtained from the naltrindole + EA + pilocarpine group significantly increased to 18.6 ± 2.3% (p < 0.05, when compared with the values obtained from the PFS + EA + pilocarpine group). The amount of REM sleep during the dark period obtained from the naltrindole + EA + pilocarpine group increased to 2.7 ± 0.6%, although it did not reach statistical significance (p > 0.05, when compared with the values obtained from the PFS + EA + pilocarpine group) (Figure 4A & 4D). The percentage of time spent in NREM sleep and REM sleep during the light period obtained from the naltrindole + EA + pilocarpine group significantly increased to 43.2 ± 1.6% (p < 0.05, when compared with the values obtained from the PFS + EA + pilocarpine group) and 9.2 ± 0.9% (p < 0.05, when compared with the values obtained from the PFS + EA + pilocarpine group), respectively (Figure 4C & 4F).

### The effect of nor-binaltorphimine on the 100 Hz EA-induced sleep alterations

Administration of *nor*-binaltorphimine also significantly blocked the 100 Hz EA-induced reductions of NREM sleep and REM sleep during both the dark period and light period (Figure 3G & 3H). The percentage of time spent in NREM sleep and REM sleep during the dark period obtained from the *nor*-binaltorphimine + EA + pilocarpine group significantly increased to 19.0 ± 2.3% (p < 0.05, when compared with the values obtained from the PFS + EA + pilocarpine group) and 5.4 ± 0.8% (p < 0.05, when compared with the values obtained from the PFS + EA + pilocarpine group), respectively (Figure 4A & 4D). The percentage of time spent in NREM sleep and REM sleep during the light period obtained from the *nor*-binaltorphimine + EA + pilocarpine group significantly increased to 33.2 ± 2.4% (p < 0.05, when compared with the values obtained from the PFS + EA + pilocarpine group) and 10.2 ± 1.1% (p < 0.05, when compared with the values obtained from the PFS + EA + pilocarpine group), respectively (Figure 4C & 4F).

## Discussion

Both temporal lobe epilepsy and status epilepticus are well established in rodents by systemic or intracerebral administration of high-dose piloparpine, a cholinergic muscarinic agonist [31]. Several brain regions, such as the amygdala, thalamus, olfactory cortex, hippocampus, neocortex, and substantial nigra, are damaged following convulsion induced by pilocarpine [31]. In fact, the interest of relation between cholinergic mechanism and epilepsy began when epileptic activities occurred in rats or mice after intraamygdaloid administration of high doses of muscarinic cholinergic agonists. Administration of bethanechol, a potent muscarinic agonist resistant to acetylcholinesterases, into amygdala develops epileptiform EEGs, which started in the amygdala and spread to the hippocampus and cortex [32]. The purpose of current study was focused on the effect of EA on focal epilepsy and its sleep disruptions, therefore, we administered low dose of pilocarpine into the CeA to induce focal epilepsy. This focal epilepsy has been validated in our preliminary study.

Acupuncture and electroacupuncture (EA) has been recommended as an alternative medicine for several therapeutic indications by the World Health Organization (WHO), such as alleviation of pain, reduction of inflammation and management of insomnia. The clinical therapeutic effects and the underlying mechanisms of EA in pain relief has been well elucidated; however, its effects in other aspects, such as neurodegenerative diseases, insomnia and epilepsy, has been less investigated. Feng-Chi acupoint (GB 20), located in the depression between the upper portion of m. sternocleidomastoideus and m. trapezius in humans, has been documented in the Lingshu Jing (the Classic of the Miraculous Pivot) and indicated the therapeutic effects in headache, dizziness, hypertension, insomnia and epilepsy. It is commonly observed that there are sleep disruptions, such as daytime somnolence and/or nighttime insomnia, in patients with epilepsy [3,4]. We previously elucidated that kindled epilepsy occurred at different zeitgeber times (ZTs) alters sleep differently; ZT0 kindled epilepsy decreases NREM sleep and REM sleep, and ZT13 kindling, on the other hand, increases NREM sleep [5,6] in experimental animals. Whereas, ZT6 kindled epilepsy alters sleep circadian rhythm [6]. Furthermore, epilepsy-induced sleep disruptions would further deteriorate and worsen the seizure control [7]. Therefore, if a therapy possesses both epilepsy suppression and improvement of sleep disturbance, it would be the most appropriate therapy for seizure control. EA stimulation of bilateral Feng-Chi acupoints may become a potential therapy to suppress epileptic activity and improve epilepsy-induced sleep disruptions according to the documentation in the Lingshu Jing. In this study, administration of pilocarpine into the CeA induced focal epilepsy and decreased both NREM sleep and REM sleep during the following light period. However, our observation demonstrated that 100 Hz EA stimulation of bilateral Feng-Chi acupoints exacerbates pilocarpine-induced focal epilepsy, suggesting no beneficial effect of EA stimulation against epileptic activity. Furthermore, we also found that 100 Hz EA stimulation of Feng-Chi acupoints *per se* induces epileptiform EEGs. The effects of high-frequency EA stimulation of Feng-Chi acupoints on epileptic induction is a specific action, because 100 Hz EA stimulation of Anmian acupoints, which are located near by the Feng-Chi acupoints, did not exhibit epileptic activity [11]. In present study, we further investigated the role of EA stimulation of bilateral Feng-Chi acupoints in normal rats and in rats with pilocarpine-induced sleep disruptions. Our results indicated that 100 Hz stimulation of bilateral Feng-Chi acupoints elicited no significant effect in the physiological sleep in normal rats, whereas EA stimuli further deteriorated pilocarpine-induced decreases of NREM sleep and REM sleep. Referring to our previous finding that 100 Hz EA stimulation of Anmian acupoints, which locate near by the Feng-Chi acupoints, enhances physiological sleep in normal rats [11], suggesting the effect of high-frequency EA of Feng-Chi acupints involves a specific mechanism. These observations, since they subvert the functions of Feng-Chi acupoints documented in the Lingshu Jing, surprise us. The possible reasons of contradiction between our findings and the documentation in Lingshu Jing are as follows. First, with or without delivering electrical currents into Feng-Chi acupoints is a fact. The effects of epileptic suppression and insomnia treatment documented in Lingshu Jing is manipulated by dry needling, whereas the exacerbation of pilocarpine-induced sleep disruptions we observed in this study was the results after EA with delivering currents into acupoints. Second, different stimulation frequencies may differ the outcomes. It is worthy to investigate the effect of different EA stimulation frequencies, especially for the lower frequency (e.g., 10 Hz), on pilocarpine-induced sleep disturbances. The last reason might be due to the outcome was measured from rats in current study, whereas the effect documented in Lingshu Jing is evaluated from humans. Nevertheless, the medical indications of the acupoints stated in the ancient Chinese literatures need to provide further scientific evidence to prove their therapeutic effects.

People might consider that both epilepsy and sleep disruption are pilocarpine-induced accompanying symptoms. It is difficult to elucidate whether sleep disturbance is due to pilocarpine *per se* or epilepsy consequence, since epilepsy always occurred when pilocarpine administered. However, our previous studies demonstrated the distinct sleep disturbances induced by amygdaloid kindling at different zeitgeber times [5,6], suggesting the role of epilepsy on sleep disruptions. Our current study elucidated that EA exacerbated sleep disturbance induced by pilocarpine, regardless of considering the cause(s) of sleep disruption.

Endogenous opioid peptides (e.g., encephalin, β-endorphin, dynorphin and endormorphin) and their receptors, such as μ-, δ- and κ-opioid receptors, mediate the most effect of acupuncture, especially in the acupuncture-induced analgesia. Han and his colleagues have revealed that low-frequency (2 Hz) EA stimuli increase met-enkephalin, but not dynorphin, in the spinal cord; while high-frequency (100 HZ) EA stimuli increase the release of dynorphin rather than that of met-enkephalin [33]. The stimulation of EA between low and high frequency (e.g. 15 Hz) activates both enkephalins and dynorphins [33]. They further demonstrated that the analgesic effect induced by low-frequency EA stimulation is mediated by μ- and/or δ-opioid receptors; in contrast, high-frequency EA-induced analgesia is mediated by κ-opioid receptors [22,23]. We have elucidated that μ-receptors in the NTS mediate the sleep enhancements induced by low-frequency (10 Hz) EA stimulation of bilateral Anmian acupoints, whereas the activation of κ-receptors contributes to the high-frequency (100 Hz) EA-induced sleep increases [10,11,30]. Because the amygdala is the epileptic focus in current animal model and plays an important role in the sleep regulation [15]–[18], we further elucidated the involvement of CeA opioid receptors in the EA-induced deterioration of sleep disruptions in rats with focal epilepsy. The dense localization of opioid receptors has been found in the amygdala [34,35]. Although drowsiness and reduced arousal are commonly observed after administration of opiates [36], morphine paradoxically increases the time spent in wakefulness and decreases the time spent in REM sleep in humans [37,38]. Oral administration of morphine or methadone significantly reduces slow wave sleep and increases stage 2 of NREM sleep [39]. Opioid receptors mediate opiate-induced sleep disruptions [40,41]. Our results demonstrated that microinjection of naloxone, naloxonazine, naltrindole or *nor*-binaltorphimine significantly blocked 100 Hz EA stimulation-induced exacerbation of sleep disruption in the rats with focal epilepsy, suggesting the role of amygdaloid opioid receptors, including the μ-receptors, δ-receptors and κ-receptors, in the effect of high-frequency EA stimulation. A strategy of employing pharmacological blockade to elucidate the involvement of particular opioid receptors in 100 Hz EA-induced sleep alteration is appropriate. However, it would be of interest to mimic the EA-induced sleep alteration by microinjection of opioid receptor agonists, e.g. β-endorphin, enkephalin and dynorphin, into the CeA in the future studies. The role of amygdala on arousal and wakefulness has been emphasized. Electrical stimulation of CeA suppresses delta wave activity in the frontal cortex and increases neocortical arousal [42]. Although our result demonstrated that administration of naloxone into CeA did not alter sleep activities in naïve rats, opioids could inhibit both excitability of pyramidal cells [43] and GABAergic interneurons [44] in the CeA. We herein hypothesized that the EA-induced exacerbation of sleep disruption may be mediated by augmented release of endogenous opiates inhibiting CeA GABAergic interneurons. However, it needs to be confirmed in future study. Nevertheless, considering together with our observation that the activation of amygdaloid opioid receptors by 100 Hz EA stimulation deteriorates pilocarpine-induced focal epilepsy, the possible reasons for 100 Hz EA of bilateral Feng-Chi acupoints to further deteriorate pilocarpine-induced sleep disruptions may be either due to the effect of EA on epilepsy exacerbation or due to the effect of EA on sleep *per se* mediated by amygdala opioid receptors, or both. It needs to be further clarified in the future study.

In order to perform the 100 Hz EA stimulation easily, rats were lightly anesthetized. Our previous and present results indicated that both NREM sleep and REM sleep during the first few hours of the dark period were decreased after rats recovered from the ketamine anesthetic. The decreases in NREM sleep and REM sleep were also observed in our previous study [10,11]. Ketamine, a cyclohexanone derivative, is used clinically as a dissociative anesthetic agent both in humans and animals. Ketamine is a noncompetitive N-methyl-D-aspartate (NMDA) receptor antagonist that blocks cation channels [45]. It has been demonstrated that administration of ketamine or MK-801, another NMDA receptor antagonist, at sub-anesthetic doses produces a robust, dose-dependent increase in the intensity of δ-power of the NREM sleep [46,47]. Furthermore, the effect of MK-801 by increasing the metabolic rate in the hippocampus and other limbic structures stimulates physiological sleep that is similar to the sleep that follows sleep deprivation, indicating the need of homeostatic recovery [48]. Therefore, the suppression of NREM and REM sleep after recovery from ketamine anesthetization during the beginning of the dark period may be due to a homeostatic compensation to the previous anesthetic state. However, this explanation needs to be further investigated.

## Conclusions

In summary, our current results indicated that high-frequency (100 Hz) EA stimulation of bilateral Feng-Chi acupoints deteriorated pilocarpine-induced sleep disruptions. The EA-induced exacerbation of sleep disruptions was blocked by administration of μ-, δ-, or κ-opioid receptor antagonist into the CeA, demonstrating the involvement of CeA opioid receptors. Our current results suggest that 100 Hz EA stimulation of bilateral Feng-Chi acupoints did not exhibit the beneficial effect against sleep disturbances in rats with focal epilepsy, whereas it further deteriorated sleep.

## Competing interests

The authors declare that they have no competing interests.

## Authors’ contributions

PLY, CYL and YFT carried out the experiments. PLY and CYL analyzed sleep data. PLY, CHC, CTL and FCC designed the experimental protocols. PLY and FCC prepared the manuscript. All authors read and approved the final manuscript.
